# A 12-immune cell signature to predict relapse and guide chemotherapy for stage II colorectal cancer

**DOI:** 10.18632/aging.103707

**Published:** 2020-08-27

**Authors:** Xianglong Tian, Xiaoqiang Zhu, Wenying Meng, Shiguang Bai, Min Shi, Shihao Xiang, Chen Zhao, Yugang Wang

**Affiliations:** 1 Department of Gastroenterology, Tongren Hospital, Shanghai Jiao Tong University School of Medicine, Shanghai, China; 2 School of Biomedical Sciences, Li Ka Shing Faculty of Medicine, The University of Hong Kong, Pok Fu Lam, Hong Kong SAR, China; 3 Department of Internal Medicine, People's Hospital of Jinping County, Yunnan Province, China

**Keywords:** colorectal cancer, immune risk score, CIBERSORT, disease-free survival, propensity score matching analysis

## Abstract

The management of stage II colorectal cancer is still difficult. We aimed to construct a new immune cell-associated signature for prognostic evaluation and guiding chemotherapy in stage II colorectal cancer. We used the “Cell Type Identification by Estimating Relative Subsets of RNA Transcripts” (CIBERSORT) method to estimate the fraction of 22 immune cells by analyzing bulk tumor transcriptomes and a LASSO Cox regression model to select the prognostic immune cells. A 12-immune cell prognostic classifier, ISCRC, was built, which could successfully discriminate the high-risk patients in the training cohort (GSE39582: HR = 3.16, 95% CI: 1.85–5.40, P < 0.0001) and another independent cohorts (GSE14333: HR = 3.47, 95% CI: 1.18–10.15, P =0.0167). The receiver operating characteristic analysis revealed that the AUC of the ISCRC model was significantly greater than that of oncotypeDX model (0.7111 versus 0.5647, p=0.0152). We introduced the propensity score matching analysis to eliminate the selection bias; survival analysis showed relatively poor prognosis after chemotherapy in stage II CRC patients. Furthermore, a nomogram was built for clinicians and did well in the calibration plots. In conclusion, this immune cell-based signature could improve prognostic prediction and may help guide chemotherapy in stage II colorectal cancer patients.

## INTRODUCTION

Colorectal cancer (CRC) is still a major health burden worldwide, it ranks as the third leading cause of cancer death, with an estimated 881,000 deaths in 2018 [1]. According to the SEER cancer statistics in 2017, the stage I/II CRC patients (Localized stage) account for about 40%, and about 35% for stage III patients (Regional stage) [2]. Surgical intervention is the basis of curative treatment for CRC, however, about 13.6% of stage II CRC patients and 21.5% of stage III CRC patients develop relapse after surgery [3]. Chemotherapy may be given after surgical resection to eradicate the remaining cancer cells. For stage III CRC, post-surgical chemotherapy is now the standard treatment [4], but the benefit of post-surgical chemotherapy remains controversial in stage II CRC. QUASAR trial reveals that chemotherapy with fluorouracil and folinic acid could improve outcomes in stage II CRC, but the absolute improvements are small, indicating that the decision to provide post-surgical chemotherapy in stage II CRC needs to be more cautious [5]. With the early screening of tumors worldwide, cancer patients are becoming younger, and more CRC patients are diagnosed in the early tumor stage [2], exacerbating the dilemma for treatment of stage II CRC patients. Thus, there is an urgent need to construct new prognostic markers in stage II CRC to discriminate the patients who may benefit from post-surgical chemotherapy.

The tumor is highly heterogeneous, as is stage II CRC. The disparities in CRC survival and the benefit of post-surgical chemotherapy may be related to the complex mechanism of tumorigenesis and development. Chromosomal instability and somatic mutation are critical genetic factors implicated in tumorigenesis [6–8]; and mis-match repair (MMR) has been extensively investigated in CRC. Studies show that CRC patients with microsatellite instability (MSI) tumors are associated with favorable outcomes, compared with the patients with microsatellite stable (MSS) tumors [9, 10]. However, MMR status cannot predict the benefits of chemotherapy in stage II CRC, nor can KRAS or BRAF mutations [11]. Recently, gene expression profiles have shown great promise in prognosis assessment. Several gene signatures have been built to evaluate patients’ prognosis, as well as predict the benefit of chemotherapy [12–17]. However, few have been applied clinically and the robustness and reliability still need further evaluation. The most widely used gene signature is the OncotypeDX colon cancer assay [18, 19], which has been commercialized since 2010 and is mainly used to predict the recurrence risk in stage II CRC [20, 21]. Thus, there is of great clinical significance to identify novel molecular markers from a new perspective.

The role of tumor-infiltrating immune cells is currently getting increasing attention in cancer research. As one of the pivotal components of the tumor microenvironment, tumor-infiltrating immune cells exert their pathophysiological functions through reciprocal communication with neoplastic cells [22, 23]. Tumor-infiltrating immune cells are consist of both mononuclear and polymorphonuclear immune cells, such as macrophages, T cells, B cells, natural killer cells, etc. Studies reveal that the abundance of tumor-infiltrating immune cells is closely related to tumor stage and has significant tumor specificity [24–26]. The prognostic value of tumor-infiltrating immune cells has also been investigated in various cancers, and the intra-tumoral γδ T-cell signatures have emerged as the most significant favorable cancer-wide prognostic populations [23, 25, 27, 28]. Besides, immune cells are also implicated in chemotherapeutic response in cancer, and macrophages are found to reduce chemotherapy sensitivity [29, 30].

Comprehensive quantification of the immune cell infiltrates in tumors can be accessed by multiple computational analyses of the gene expression profiles, which are relatively easy to obtain from the accumulating public microarray data and RNA sequencing data of human tumors [31]. The algorithm of “Cell Type Identification by Estimating Relative Subsets of RNA Transcripts” (CIBERSORT) is the current most accurate and extensively used computational algorithm available for enumeration of various immune cell types [32, 33]. In our study, CIBERSORT was used to assess the distribution of 22 immune cells from tumor RNA transcripts, and then the least absolute shrinkage and selection operator method (LASSO) method was introduced to construct a 12-immune cell signature to predict disease-free survival (DFS) and assist in evaluating the benefit of chemotherapy for stage II CRC patients.

## RESULTS

### Development and validation of the ISCRC model

**Figure 1:**
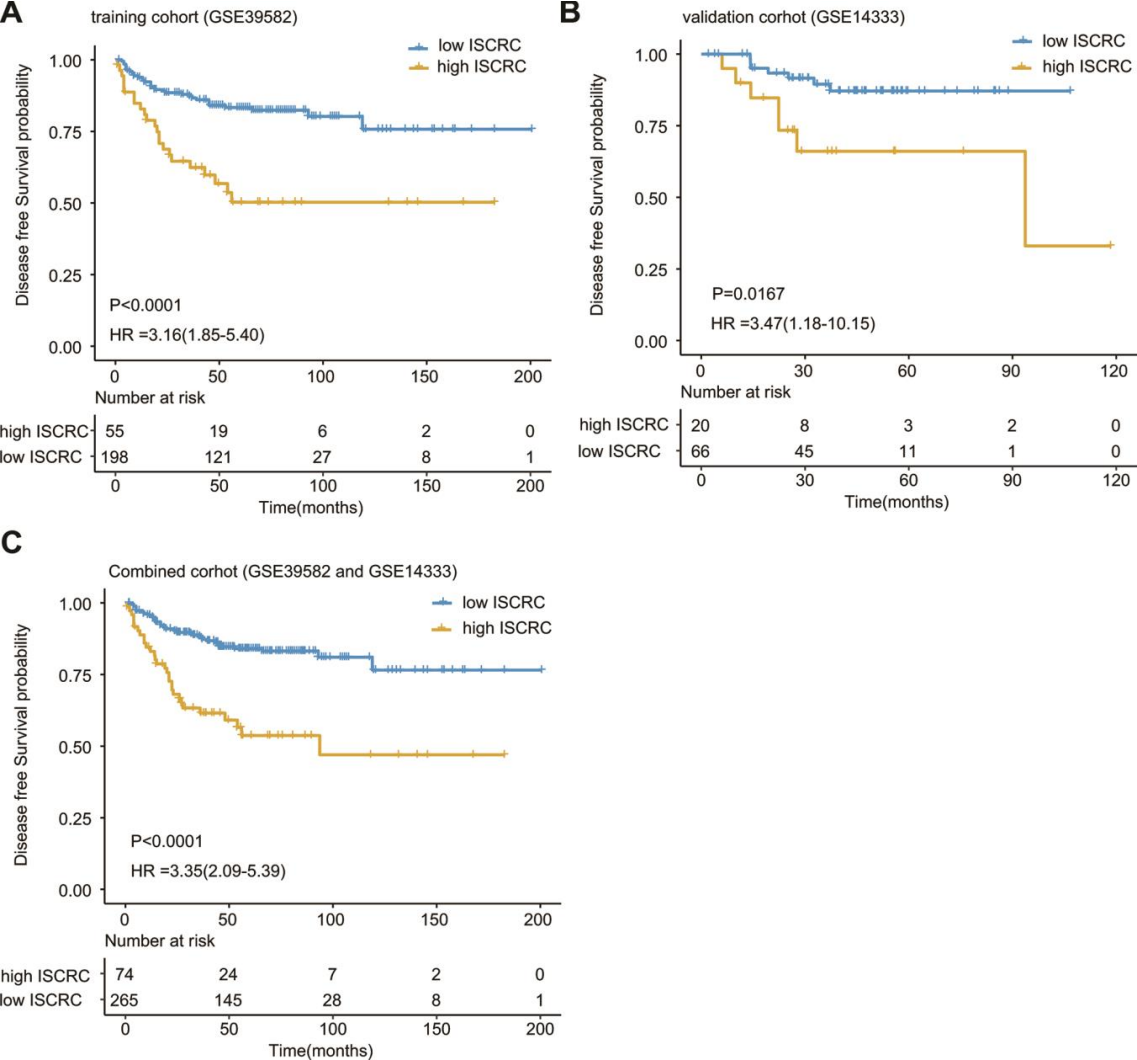
**Kaplan-Meier estimates of the patients’ DFS using the ISCRC model.** The Kaplan-Meier plots were used to visualize the patients’ recurrence probabilities for the low-ISCRC versus high-ISCRC group of patients from corresponding GEO datasets. (**A**) Kaplan-Meier curves for training dataset GSE39582 (N=253); (**B**) Kaplan-Meier curves for GSE14333 (N=86); (**C**) Kaplan-Meier curves for combined dataset (GSE39582 and GSE14333) (N=339). The tick marks on the Kaplan-Meier curves represent the censored subjects. The differences between the two curves were determined by the two-side log-rank test.

The LASSO Cox regression was introduced to interrogate the relevance between 22 immune cells and the patients’ survival in the training cohort-GSE39582, (Supplementary file 1 and Supplementary Figure 1), and a 12-immune cell model was identified, which was significantly associated with DFS of stage II CRC. The 12 immune cells and associated coefficients generated through LASSO analysis were shown in Supplementary file 2 and Supplementary Table 1. The immune scores for CRC patients, namely ISCRC, were calculated based on the fractions of 12 immune cells in each sample and the associated coefficients, and the formula of calculation was shown in Supplementary files 3 and Supplementary Table 2. A dichotomous ISCRC was adopted in survival analysis, based on the optimum cut-off value of ISCRC (0.7473), patients were divided into low- and high-ISCRC groups. The Kaplan-Meier survival analysis revealed that patients in the high-ISCRC group had worse outcomes compared with the low-ISCRC group [hazard ratio (HR) =3.16, 95% confidence interval (CI) =1.85-5.40, *P* < 0.0001; Figure 1A]. The efficacy of the ISCRC model for the prognosis prediction in stage II CRC patients was further validated in another independent dataset GSE14333. Patients were also classified into two subgroups using the same cut-off value (0.7473), and it generated consistent results (HR=3.47, 95% CI=1.18-10.15, *P* = 0.0167, Figure 1B). We also validated the ISCRC model in the combined cohort of GSE39852 and GSE14333, and a significantly different prognosis can be seen between low- and high-ISCRC groups (HR=3.35, 95% CI=2.09-3.93, *P* < 0.0001, Figure 1C).

**Figure 2:**
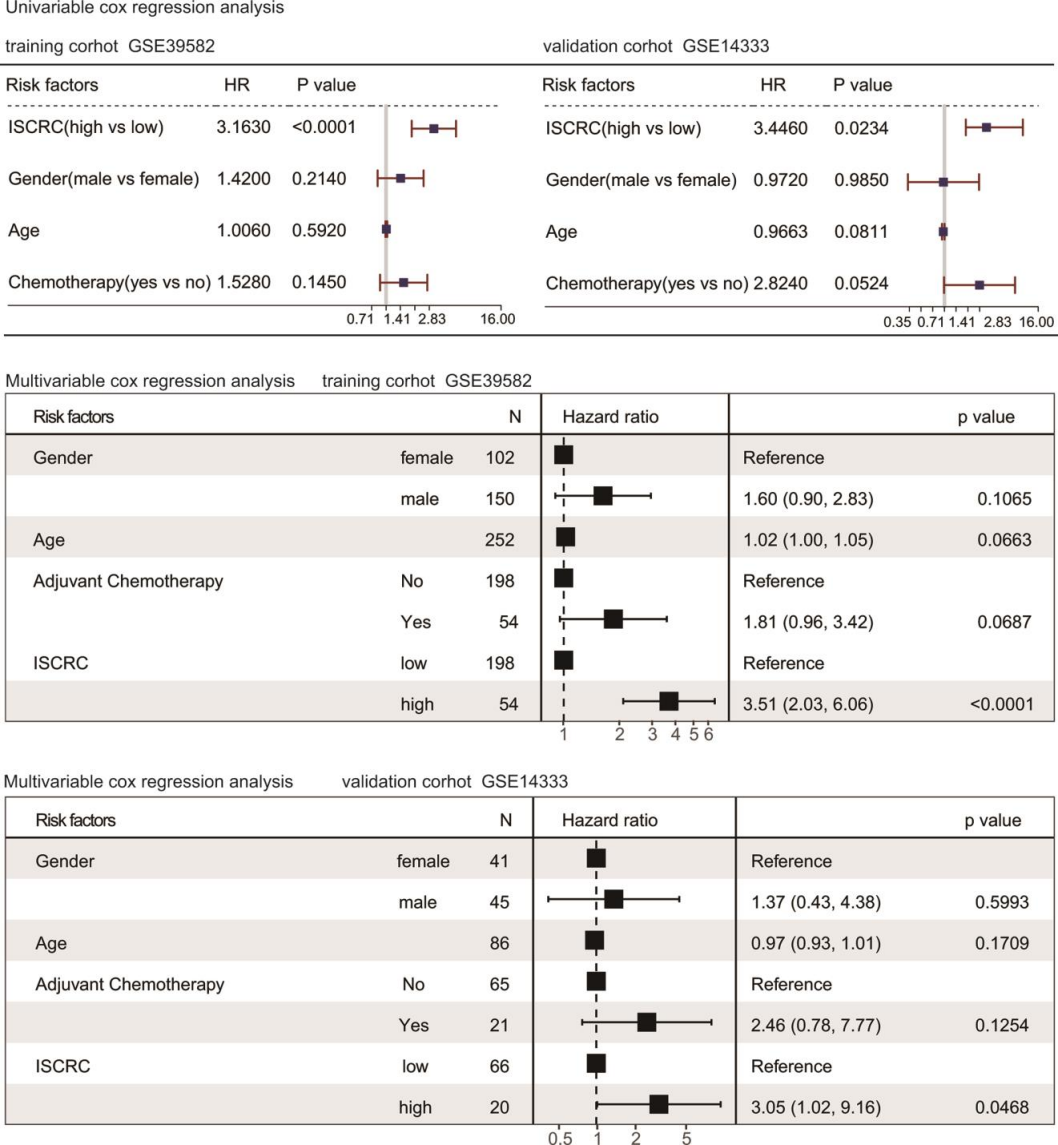
**Forest plot summary of analyses of disease-free survival (DFS).** Univariable and multivariable analyses of the ISCRC model, age, gender, and chemotherapy on GSE39582 and GSE14333 datasets. The blue squares on the transverse lines represent the hazard ratio (HR), and the red transverse lines represent 95% CI. ISCRC and age are continuous variables, gender and chemotherapy are discontinuous variables.

In the univariate Cox regression model, the ISCRC classifier was found to be a strong variable correlated with CRC recurrence in both training and validation cohorts (GSE39582: HR=3.1630, *P* <0.0001; GSE14333: HR=3.4460, *P* = 0.0234, Figure 2A). After adjustment for the common clinical covariates, including age, sex, and chemotherapy, multivariate Cox regression analysis demonstrated that the ISCRC classifier remained an independent prognostic factor for DFS in the training dataset (GSE39582: HR=3.51, 95%CI=2.03-6.06, *P* < 0.0001; Figure 2B) and the validation dataset (GSE14333: HR=3.05, 95%CI=1.02-9.16, *P* = 0.0468; Figure 2C). To investigate the sensitivity and specificity of survival prediction, the receiver operating characteristic (ROC) analysis was also performed to calculate the area under ROC curve (AUC) of the ISCRC model. Supplementary file 4 and Supplementary Figure 2 showed that our ISCRC model owned considerable predicted power of prognostic evaluation for stage II CRC patients in the training cohort (GSE39582: AUC=0.7111) and the validation dataset (GSE14333: AUC= 0.7041).

### Comparison ISCRC with OncotypeDX colon

**Figure 3:**
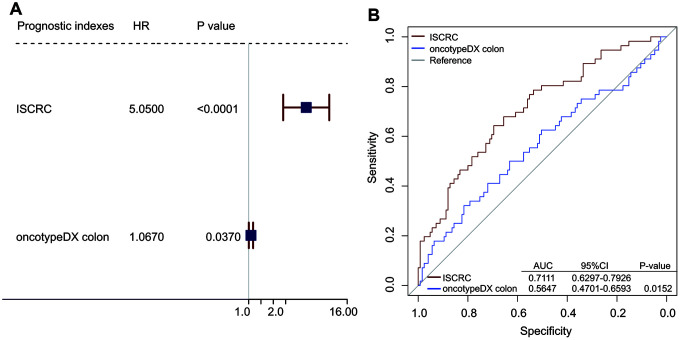
**Comparison analyses between ISCRC model and other known gene maker-oncotypeDX colon.** (**A**) Univarible analyses of the ISCRC, and oncotypeDX colon to investigate the association between each prognostic index and DFS using the prognostic indexes as continuous variables in GSE39582. The blue squares on the transverse lines represent the hazard ratio (HR), and the red transverse lines represent 95% CI. (**B**) Receiver operating characteristic (ROC) analysis of the sensitivity and specificity of the recurrence prediction by the ISCRC model and oncotypeDX colon in GSE14333.

To further evaluate the prognostic value of the ISCRC model, comparison analysis was performed between our ISCRC model and other known gene makers. We did not mean to make a comprehensive review of all prognostic biomarkers in CRC, thus the widely used gene signature, oncotypeDX colon cancer assay, was selected as the representative. The prognostic indexes of the oncotypeDX model were calculated according to the associated formula (See Supplementary file 3 and Supplementary Table 2). We first performed the univariable Cox regression analysis in GSE39582 to assess the prognostic value, where the prognostic index was used as a continuous variable. As shown in Figure 3A, the ISCRC and oncotypeDX models were all significantly associated with DFS in stage II CRC, but the HR of our ISCRC model was significantly larger, with an even lower p-value. ROC analysis was also performed in GSE39582 to assess the sensitivity and specificity of survival prediction. As shown in Figure 3B, the AUC of the ISCRC model was significantly greater than that of the oncotypeDX model (0.7111 versus 0.5647, p=0.0152), indicating well prognostic accuracy of our ISCRC model.

### ISCRC and the benefit of chemotherapy

**Figure 4:**
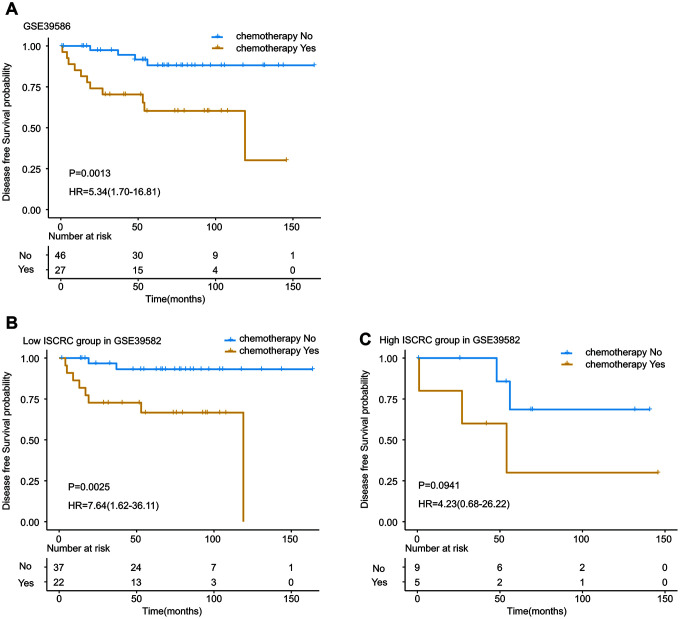
**Kaplan-Meier estimates of the effect of adjuvant chemotherapy on patients’ DFS.** To eliminate the selection bias, propensity score matching analysis was performed between chemotherapy and non-chemotherapy groups. The Kaplan-Meier plots were used to visualize the patients’ recurrence probabilities for chemotherapy versus non-chemotherapy group of patients in GSE39582. (**A**) Kaplan-Meier curves for total GSE39582 dataset (N=73); (**B**) Kaplan-Meier curves for low ISCRC patients in GSE39582 (N=59); (**C**) Kaplan-Meier curves for high ISCRC patients in GSE39582 (N=14). The tick marks on the Kaplan-Meier curves represent the censored subjects. The differences between the two curves were determined by the two-side log-rank test.

Studies have revealed the close association between chemotherapy and tumor microenvironment. One of the crucial components of the tumor microenvironment is the immune cell, and macrophages have been reported to promote tumor angiogenesis and drive chemotherapy resistance [29, 30, 34]. In our study, the ISCRC model demonstrated well prognostic value for stage II CRC patients, so we speculated that our immune cell-derived prognostic biomarker might be associated with chemosensitivity. All the two datasets in our study provided information on chemotherapy, so we intended to examine the benefit of chemotherapy in stage II patients. As a random assignment of chemotherapy to samples is not feasible in the retrospective study, the presence of an imbalance in baseline characteristics between the control and treatment groups can lead to a biased estimation [35]. Thus, the propensity score matching analysis was performed to eliminate the selection bias. After propensity score matching analysis, 82 matched samples were generated in GSE39582, but only 16 matched samples left in GSE14333, so the cohort GSE14333 was not suitable for further analysis (see supplementary file 5 and Supplementary Table 3). The matched patients in GSE39582 were classified into two subgroups based on the status of chemotherapy, as shown in Figure 4A, Kaplan-Meier survival analysis revealed that the prognosis of the patients in chemotherapy group was significantly worse than in non-chemotherapy group (HR=5.34,95%CI=1.70-16.81, p=0.0013). We further investigated the effect of chemotherapy in low- and high-ISCRC groups, respectively. Figure 4B demonstrated the similar result in the low-ISCRC group (HR=7.64, 95%CI=1.62-36.11, P=0.0025), however, no significant difference was seen in the high-ISCRC group (HR=4.23, 95%CI=0.68-26.22, p=0.0941) (Figure 4C), these results suggested that stage II patients in the low-ISCRC group were not suitable for chemotherapy.

### Distribution of ISCRC and clinical characteristics

**Figure 5:**
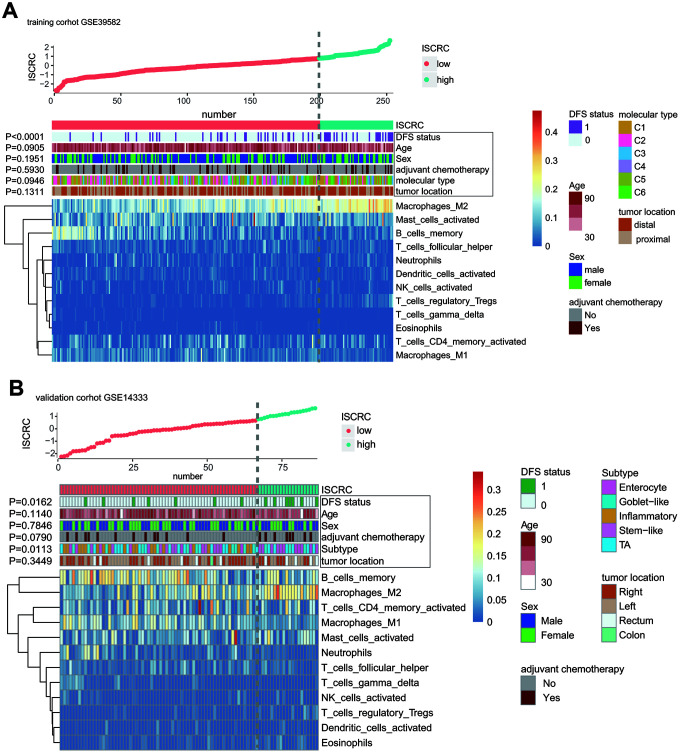
**The correlation between immune risk score (namely ISCRC model) and other clinicopathological characteristics in GSE39582 and GSE14333.** The distribution of ISCRC, patients’ recurrence status, age, sex, adjuvant chemotherapy, molecular type, tumor location, and immune cells were analyzed in the GSE39582 (**A**) and GSE14333 (**B**).

Our ISCRC model was composed of 12 immune cells, and then the distribution of 12 immune cells and the common clinical parameters in the low- and high-ISCRC groups was illustrated in Figure 5. Our study revealed that the low-ISCRC group was characterized by high expression of M1 Macrophages and B memory cells but low expression of M2 Macrophages, while the distribution of these three immune cells in the high-ISCRC group was opposite in training cohort GSE39582 (Figure 5A). A similar distribution characteristic can also be observed in GSE14333 (Figure 5B). The DFS status was significantly different between the low- and high-ISCRC groups in both GSE38582 and GSE14333, and there were more recurred patients in the high-ISCRC group. However, the distribution of the common clinical factors between different ISCRC groups did not vary significantly.

### Construction of nomogram based on ISCRC

**Figure 6:**
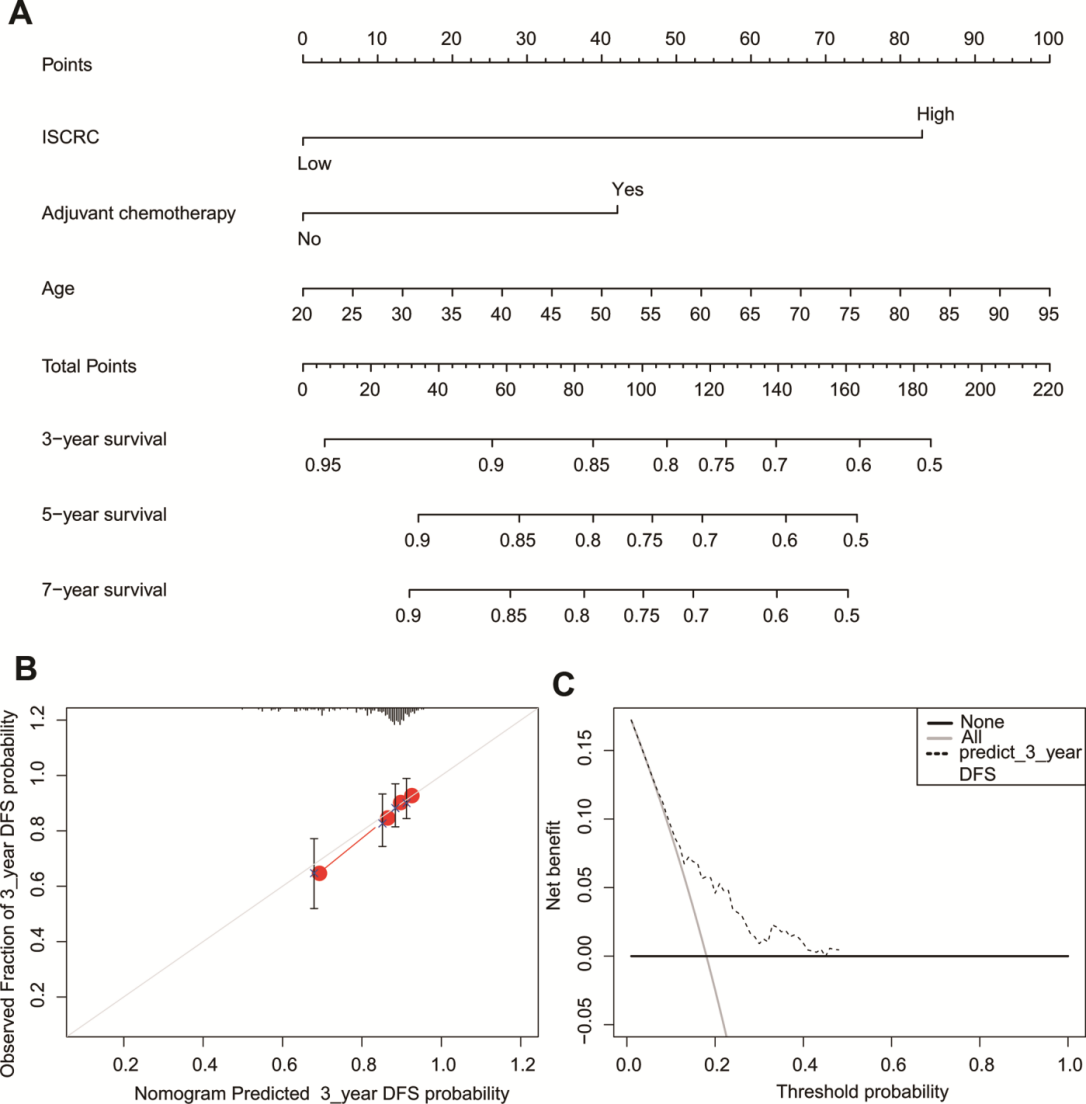
**The nomogram to predict risk of cancer recurrence in GSE39582.** (**A**) The nomogram for predicting proportion of patients with disease-free survival. (**B**) The calibration plots for predicting recurrence at 3 years. Nomogram-predicted probability of recurrence is plotted on the x-axis; actual recurrence is plotted on the y-axis. The solid line represents our nomogram and the vertical bars represent 95 percent confidence intervals. (**C**) ROC curve based on the nomogram for recurrence probability.

To develop a quantitative recurrence prediction method for clinical application, a nomogram was built, which integrated the ISCRC model, age, sex, and chemotherapy status (Figure 6A). The nomogram revealed that age had the largest impact on the patients’ prognosis, followed by the ISCRC model and adjuvant chemotherapy. The total points for each patient were calculated based on these variables to estimate the possibility of recurrence in the following 3-, 5- and 7-year for each stage II CRC patient. The performance of the nomogram was evaluated by calibration plots (Figure 6B). The line-segment was close to 45-degree, suggesting that the nomogram did quite well. DCA showed a well clinical utility of the nomogram (Figure 6C).

## DISCUSSION

Recently, the study of the immune contexture has receiving accumulating attention in cancer research [24, 36]. Several immune score models have been developed from analyzing the fractions of immune cells to predict prognosis in various types of tumors, including CRC [28, 37, 38]. However, the immune score model, especially for stage II CRC, has not been reported yet. In our study, we have constructed a novel immune cell-derived prognostic marker, ISCRC model, to predict the relapse of stage II CRC. The prognostic value of the ISCRC model was confirmed in an independent validation cohort and the combined cohort, indicating good reproducibility. When compared with the representative known gene signature-oncotypeDX colon, the ISCRC model exhibited a better predictive capability for DFS, so we concluded that our ISCRC model could improve the recurrence prediction in stage II CRC. Furthermore, our study demonstrated the adverse effect of chemotherapy in stage II CRC, especially for patients with low-ISCRC. This finding might shed new light on the eligibility of adjuvant chemotherapy for stage II CRC patients.

Immune cells are implicated in the prognosis of cancer patients, and the underlying mechanism is partly due to the immune-suppression effect induced by tumor-associated dendritic cells, neutrophil, and M2 macrophages, etc. [25, 39–41]. However, it is very difficult to measure the immune infiltrates in tumors by traditional methods, such as IHC or flow cytometry; they are not qualified for the task of discriminating varieties of immune cells and quantifying them simultaneously. It is well known that solid tumors are highly heterogeneous and lack distinctive markers for various cells. Therefore, a new method is urgently needed to comprehensively study the immune contexture in cancers. In our study, a newly developed computer-based analytical algorithm, CIBERSORT, was introduced to evaluate the fractions of immune cells, which allows estimating the abundance of immune cells simultaneously in a large patient cohort with high accuracy. Then the LASSO analysis was performed to construct the immune cell-derived prognostic model-ISCRC; it is a reliable instrument in selecting the prognostic markers and has been extensively used in high-dimensional microarray data [42–44]. We validated the ISCRC model in another independent cohort-GSE14333. Furthermore, comparison analysis showed that the ISCRC model had better predictive power compared with the representative gene signature-oncotypeDX, which has been used clinically and validated in a large prospective study [20]. These findings indicated that our ISCRC model could improve the prognostic prediction in stage II CRC with fair reliability.

Interestingly, we found that the distribution of common clinical factors was not significantly different between low- and high-ISCRC groups in stage II CRC patients, so the ISCRC model might serve as a good supplement to the clinical-pathological features for stage II CRC. Further analysis revealed that the low-ISCRC group was characterized by high expression of M1 Macrophages and low expression of M2 Macrophages, while the high-ISCRC group presented the opposite distribution feature.

Macrophages play a pivotal role in innate immunity and are closely associated with inflammation and host defense [45]. The patterns of macrophage activation can roughly divide into two main types: classically activated macrophages (M1) and alternatively activated macrophages (M2). The M1-M2 macrophages are characterized by distinctive secretome profile and biological functions [46]. M1 Macrophages can promote Th1 response and show strong antineoplastic activity [47]. M2 Macrophages are implicated in immunosuppression, chronic inflammation, etc. [41]; M2 macrophage polarization can promote tumor progression [48, 49]. In our study, the high-ISCRC group showed poor prognosis compared with the low-ISCRC group. The potential mechanism might partly attribute to the immune characteristics of our ISCRC model; the high-ISCRC group was found to be characterized by high expression of M2 macrophages and low expression of M1 macrophages, thus resulting in a poor outcome.

Adjuvant chemotherapy is of great significance in cancer treatment. However, the rationality of chemotherapy in stage II CRC remains controversial. Up to date, there is still some conflicting evidence in clinical trials [50, 51]. The different responses to chemotherapy in stage II patients might attribute to the inherent heterogeneity of the tumor. The significant difference of immune contexture can be seen between the low- and high-ISCRC groups, although with the same TNM stage. Our study demonstrated that adjuvant chemotherapy might exert adverse effects on stage II patients, especially for patients with low-ISCRC, so chemotherapy in patients with stage II CRC needs to be more cautious. Notably, when we interrogated the prognosis between the low- and high-ISCRC groups, the propensity score matching method was introduced to remove the selection bias, which has been widely used for retrospective analysis [52, 53]. In our study, the potential confounding factors, such as patients’ age, etc., were balanced in the two subgroups to accurately weigh the risk-benefit ratio of chemotherapy in stage II CRC. These findings indicated that the ISCRC model might be a useful biomarker to predict the patients’ prognosis and guide chemotherapy in stage II CRC.

There are some limitations to our study. First, our study is with limited sample size, and the ratio of patients with chemotherapy to non-chemotherapy patients is relatively low in the two datasets. After propensity score matching, the sample size of GSE14333 becomes even smaller, which is not suitable for further analysis. Second, it is a retrospective analysis using publicly available datasets. The detailed clinical information for each patient is unavailable; the selection bias is hard to avoid, although propensity score matching has been performed. Thus, the reliability of our analysis requires to be further verified in a prospective study.

## CONCLUSIONS

In our study, a new immune cell-related marker was built through investigating the whole immune landscape to predict prognosis and evaluate the benefit of post-surgical chemotherapy in stage II CRC. The prognostic value of the ISCRC model was confirmed in another independent cohort and the combined cohort. Moreover, our study demonstrated the adverse effect of chemotherapy in stage II CRC patients, especially for patients with low-ISCRC. Thus, this analysis might have a beneficial effect on the personalized management of stage II CRC patients.

## MATERIALS AND METHODS

### CRC datasets preparation

CRC RNA transcripts data and corresponding clinical information in our study were downloaded from ArrayExpress (http://www.ebi.ac.uk/arrayexpress/) and Gene Expression Omnibus (GEO) (https://www.ncbi.nlm.nih.gov/geo/). The raw files of microarray data were normalized using a robust multi-array averaging method [54], and the process of which was performed with “affy” and “affycoretools” packages of R software. A total of 339 CRC stage II patients were selected from two datasets for this study, that were GSE39582 (n=253) and GSE14333 (n=86). Dukes’ B CRC patients in the GSE14333 cohort were used for our analyses, which are corresponding to the American Joint Committee on Cancer (AJCC) stage II patients based on AJCC Colon and Rectum Cancer staging 7^th^ Edition.

### Estimation of the immune cell type fractions through CIBERSORT

For estimation of the abundance of immune cells in CRC cancer samples, the gene expression data were processed through the CIBERSORT web portal (http://cibersort.stanford.edu/). The immune cells are distinguished with a leukocyte gene signature matrix, termed LM22. The 22 human hematopoietic cells are grouped into seven T cell types, naïve and memory B cells, plasma cells, NK cells, and myeloid subsets, and the cell subsets can be further divided into 11 major leukocyte types [32]. The CIBERSORT method can discriminate the human immune cells with high sensitivity and specificity and has been well validated on microarray data. The estimated fractions of immune cell subsets by this method are considered to be accurate with the threshold of P <0.05 [33, 55]. The estimated CIBERSORT results were normalized and the sum of proportions of 22 immune cells in each sample was equal to 1.

### Construction and validation of the ISCRC model

GSE39582 dataset was used as the training cohort to perform the LASSO Cox regression analysis with the “glmnet” package of R software (version 3.5.1) (R Foundation for Statistical Computing, Vienna, and Austria). The penalized Cox regression model with LASSO penalty can achieve shrinkage and variable selection simultaneously with 10-times cross-validations [44, 56]. Based on the optimal penalty parameter lambda, the most useful prognostic markers with associated coefficients were screened out from the 22 candidate immune cells. The immune score for each sample in CRC was calculated based on the fraction of each immune cell and its associated coefficient, then they were normalized and the “survminer” package was used to determine the best cut-off value. Thus, a multi-immune cell classifier, ISCRC, was constructed to predict the recurrence probability for CRC patients. All samples were split into low-ISCRC and high-ISCRC groups, and the prognostic value of ISCRC was further validated in GSE14333. The workflow of this study was depicted in Supplementary file 6 and Supplementary Figure 3.

### Statistical analysis

DFS was from the time of surgical intervention to the first confirmed relapse. The DFS differences were estimated by the Kaplan-Meier estimator and the log-rank test. To investigate the effect of chemotherapy on prognosis, the propensity matching analysis was conducted with the “MatchIt” package to remove the selection bias and the “exact” method was used to match samples between the chemotherapy group and the non-chemotherapy group [57].

The correlation between our ISCRC model and clinicopathological features was assessed using the chi-square test and one-way ANOVA test. Univariate and multivariate Cox proportional hazards regression models were performed to estimate the prognostic value of ISCRC and clinical characteristics.

To assess the sensitivity and specificity of the survival prediction, ROC analysis was performed to calculate the AUC with the “pROC” package, which served as a measure to assess the accuracy of diagnosis [58]; the method “delong” was used to investigate the differences between ROC curves. For ROC analysis, the samples were excluded who still did not relapse at last follow-up and whose follow-up times were relatively short (less than the median DFS) [59].

The nomogram was constructed using Cox regression coefficients of ISCRC and other clinical factors and the nomogram plots were generated with the “rms” R package. The performance characteristics of the nomogram were assessed by calibration plots, where the 45-degree line stood for the ideal prediction model. Decision curve analysis (DCA) was introduced to explore the clinical utility of the nomogram [60, 61]. All the statistical analyses in our study were performed with R software (3.5.1) and a p-value less than 0.05 was regarded to be statistically significant.

## Supplementary Material

Supplementary Figures

Supplementary Tables

## References

[1] Bray F, Ferlay J, Soerjomataram I, Siegel RL, Torre LA, Jemal A. Global cancer statistics 2018: GLOBOCAN estimates of incidence and mortality worldwide for 36 cancers in 185 countries. CA Cancer J Clin. 2018; 68:394–424. 10.3322/caac.21492 30207593

[2] Siegel RL, Miller KD, Fedewa SA, Ahnen DJ, Meester RG, Barzi A, Jemal A. Colorectal cancer statistics, 2017. CA Cancer J Clin. 2017; 67:177–93. 10.3322/caac.21395 28248415

[3] Böhm B, Schwenk W, Hucke HP, Stock W. Does methodic long-term follow-up affect survival after curative resection of colorectal carcinoma? Dis Colon Rectum. 1993; 36:280–86. 10.1007/BF02053511 8449134

[4] Adjuvant therapy for patients with colon and rectal cancer. National institutes of health. Conn Med. 1990; 54:573–81. 2265546

[5] Gray R, Barnwell J, McConkey C, Hills RK, Williams NS, Kerr DJ, and Quasar Collaborative Group. Adjuvant chemotherapy versus observation in patients with colorectal cancer: a randomised study. Lancet. 2007; 370:2020–29. 10.1016/S0140-6736(07)61866-2 18083404

[6] Pino MS, Chung DC. The chromosomal instability pathway in colon cancer. Gastroenterology. 2010; 138:2059–72. 10.1053/j.gastro.2009.12.065 20420946 PMC4243705

[7] Sparks AB, Morin PJ, Vogelstein B, Kinzler KW. Mutational analysis of the APC/beta-catenin/tcf pathway in colorectal cancer. Cancer Res. 1998; 58:1130–34. 9515795

[8] Malumbres M, Barbacid M. RAS oncogenes: the first 30 years. Nat Rev Cancer. 2003; 3:459–65. 10.1038/nrc1097 12778136

[9] Popat S, Hubner R, Houlston RS. Systematic review of microsatellite instability and colorectal cancer prognosis. J Clin Oncol. 2005; 23:609–18. 10.1200/JCO.2005.01.086 15659508

[10] Watanabe T, Wu TT, Catalano PJ, Ueki T, Satriano R, Haller DG, Benson AB 3rd, Hamilton SR. Molecular predictors of survival after adjuvant chemotherapy for colon cancer. N Engl J Med. 2001; 344:1196–206. 10.1056/NEJM200104193441603 11309634 PMC3584633

[11] Hutchins G, Southward K, Handley K, Magill L, Beaumont C, Stahlschmidt J, Richman S, Chambers P, Seymour M, Kerr D, Gray R, Quirke P. Value of mismatch repair, KRAS, and BRAF mutations in predicting recurrence and benefits from chemotherapy in colorectal cancer. J Clin Oncol. 2011; 29:1261–70. 10.1200/JCO.2010.30.1366 21383284

[12] Agesen TH, Sveen A, Merok MA, Lind GE, Nesbakken A, Skotheim RI, Lothe RA. ColoGuideEx: a robust gene classifier specific for stage II colorectal cancer prognosis. Gut. 2012; 61:1560–67. 10.1136/gutjnl-2011-301179 22213796

[13] O’Connell MJ, Lavery I, Yothers G, Paik S, Clark-Langone KM, Lopatin M, Watson D, Baehner FL, Shak S, Baker J, Cowens JW, Wolmark N. Relationship between tumor gene expression and recurrence in four independent studies of patients with stage II/III colon cancer treated with surgery alone or surgery plus adjuvant fluorouracil plus leucovorin. J Clin Oncol. 2010; 28:3937–44. 10.1200/JCO.2010.28.9538 20679606 PMC2940392

[14] van der Stok EP, Smid M, Sieuwerts AM, Vermeulen PB, Sleijfer S, Ayez N, Grünhagen DJ, Martens JW, Verhoef C. mRNA expression profiles of colorectal liver metastases as a novel biomarker for early recurrence after partial hepatectomy. Mol Oncol. 2016; 10:1542–50. 10.1016/j.molonc.2016.09.002 27692894 PMC5423127

[15] Oh SC, Park YY, Park ES, Lim JY, Kim SM, Kim SB, Kim J, Kim SC, Chu IS, Smith JJ, Beauchamp RD, Yeatman TJ, Kopetz S, Lee JS. Prognostic gene expression signature associated with two molecularly distinct subtypes of colorectal cancer. Gut. 2012; 61:1291–98. 10.1136/gutjnl-2011-300812 21997556 PMC3419333

[16] Jensen NF, Stenvang J, Beck MK, Hanáková B, Belling KC, Do KN, Viuff B, Nygård SB, Gupta R, Rasmussen MH, Tarpgaard LS, Hansen TP, Budinská E, et al. Establishment and characterization of models of chemotherapy resistance in colorectal cancer: towards a predictive signature of chemoresistance. Mol Oncol. 2015; 9:1169–85. 10.1016/j.molonc.2015.02.008 25759163 PMC5528766

[17] Schell MJ, Yang M, Missiaglia E, Delorenzi M, Soneson C, Yue B, Nebozhyn MV, Loboda A, Bloom G, Yeatman TJ. A composite gene expression signature optimizes prediction of colorectal cancer metastasis and outcome. Clin Cancer Res. 2016; 22:734–45. 10.1158/1078-0432.CCR-15-0143 26446941 PMC4802496

[18] Clark-Langone KM, Sangli C, Krishnakumar J, Watson D. Translating tumor biology into personalized treatment planning: analytical performance characteristics of the oncotype DX colon cancer assay. BMC Cancer. 2010; 10:691. 10.1186/1471-2407-10-691 21176237 PMC3016296

[19] Webber EM, Lin JS, Whitlock EP. Oncotype DX tumor gene expression profiling in stage II colon cancer. Application: prognostic, risk prediction. PLoS Curr. 2010; 2:RRN1177. 10.1371/currents.RRN1177 20877447 PMC2940137

[20] Srivastava G, Renfro LA, Behrens RJ, Lopatin M, Chao C, Soori GS, Dakhil SR, Mowat RB, Kuebler JP, Kim G, Mazurczak M, Lee M, Alberts SR. Prospective multicenter study of the impact of oncotype DX colon cancer assay results on treatment recommendations in stage II colon cancer patients. Oncologist. 2014; 19:492–97. 10.1634/theoncologist.2013-0401 24710310 PMC4012966

[21] Brenner B, Geva R, Rothney M, Beny A, Dror Y, Steiner M, Hubert A, Idelevich E, Gluzman A, Purim O, Shacham-Shmueli E, Shulman K, Mishaeli M, et al. Impact of the 12-gene colon cancer assay on clinical decision making for adjuvant therapy in stage II colon cancer patients. Value Health. 2016; 19:82–87. 10.1016/j.jval.2015.08.013 26797240

[22] Korneev KV, Atretkhany KN, Drutskaya MS, Grivennikov SI, Kuprash DV, Nedospasov SA. TLR-signaling and proinflammatory cytokines as drivers of tumorigenesis. Cytokine. 2017; 89:127–35. 10.1016/j.cyto.2016.01.021 26854213

[23] Hanahan D, Coussens LM. Accessories to the crime: functions of cells recruited to the tumor microenvironment. Cancer Cell. 2012; 21:309–22. 10.1016/j.ccr.2012.02.022 22439926

[24] Fridman WH, Pagès F, Sautès-Fridman C, Galon J. The immune contexture in human tumours: impact on clinical outcome. Nat Rev Cancer. 2012; 12:298–306. 10.1038/nrc3245 22419253

[25] Gentles AJ, Newman AM, Liu CL, Bratman SV, Feng W, Kim D, Nair VS, Xu Y, Khuong A, Hoang CD, Diehn M, West RB, Plevritis SK, Alizadeh AA. The prognostic landscape of genes and infiltrating immune cells across human cancers. Nat Med. 2015; 21:938–45. 10.1038/nm.3909 26193342 PMC4852857

[26] Becht E, Giraldo NA, Dieu-Nosjean MC, Sautès-Fridman C, Fridman WH. Cancer immune contexture and immunotherapy. Curr Opin Immunol. 2016; 39:7–13. 10.1016/j.coi.2015.11.009 26708937

[27] Engblom C, Pfirschke C, Zilionis R, Da Silva Martins J, Bos SA, Courties G, Rickelt S, Severe N, Baryawno N, Faget J, Savova V, Zemmour D, Kline J, et al. Osteoblasts remotely supply lung tumors with cancer-promoting SiglecF^high^ neutrophils. Science. 2017; 358:eaal5081. 10.1126/science.aal5081 29191879 PMC6343476

[28] Pagès F, Mlecnik B, Marliot F, Bindea G, Ou FS, Bifulco C, Lugli A, Zlobec I, Rau TT, Berger MD, Nagtegaal ID, Vink-Börger E, Hartmann A, et al. International validation of the consensus immunoscore for the classification of colon cancer: a prognostic and accuracy study. Lancet. 2018; 391:2128–39. 10.1016/S0140-6736(18)30789-X 29754777

[29] Salvagno C, Ciampricotti M, Tuit S, Hau CS, van Weverwijk A, Coffelt SB, Kersten K, Vrijland K, Kos K, Ulas T, Song JY, Ooi CH, Rüttinger D, et al. Therapeutic targeting of macrophages enhances chemotherapy efficacy by unleashing type I interferon response. Nat Cell Biol. 2019; 21:511–21. 10.1038/s41556-019-0298-1 30886344 PMC6451630

[30] Halbrook CJ, Pontious C, Kovalenko I, Lapienyte L, Dreyer S, Lee HJ, Thurston G, Zhang Y, Lazarus J, Sajjakulnukit P, Hong HS, Kremer DM, Nelson BS, et al. Macrophage-released pyrimidines inhibit gemcitabine therapy in pancreatic cancer. Cell Metab. 2019; 29:1390–99.e6. 10.1016/j.cmet.2019.02.001 30827862 PMC6602533

[31] Finotello F, Trajanoski Z. Quantifying tumor-infiltrating immune cells from transcriptomics data. Cancer Immunol Immunother. 2018; 67:1031–40. 10.1007/s00262-018-2150-z 29541787 PMC6006237

[32] Newman AM, Liu CL, Green MR, Gentles AJ, Feng W, Xu Y, Hoang CD, Diehn M, Alizadeh AA. Robust enumeration of cell subsets from tissue expression profiles. Nat Methods. 2015; 12:453–57. 10.1038/nmeth.3337 25822800 PMC4739640

[33] Fu H, Zhu Y, Wang Y, Liu Z, Zhang J, Xie H, Fu Q, Dai B, Ye D, Xu J. Identification and Validation of Stromal Immunotype Predict Survival and Benefit from Adjuvant Chemotherapy in Patients with Muscle-Invasive Bladder Cancer. Clin Cancer Res. 2018; 24:3069–3078. 10.1158/1078-0432.CCR-17-2687 29514839

[34] Zhang L, Qi Y, Min H, Ni C, Wang F, Wang B, Qin H, Zhang Y, Liu G, Qin Y, Duan X, Li F, Han X, et al. Cooperatively Responsive Peptide Nanotherapeutic that Regulates Angiopoietin Receptor Tie2 Activity in Tumor Microenvironment To Prevent Breast Tumor Relapse after Chemotherapy. ACS Nano. 2019; 13:5091–5102. 10.1021/acsnano.8b08142 30986342

[35] D’Agostino RB Jr. Propensity score methods for bias reduction in the comparison of a treatment to a non-randomized control group. Stat Med. 1998; 17:2265–81. 10.1002/(sici)1097-0258(19981015)17:19<2265::aid-sim918>3.0.co;2-b 9802183

[36] Sidaway P. Immunoscore provides a more accurate prognosis. Nat Rev Clin Oncol. 2018; 15:471. 10.1038/s41571-018-0050-y 29872178

[37] Ogino S, Giannakis M. Immunoscore for (colorectal) cancer precision medicine. Lancet. 2018; 391:2084–86. 10.1016/S0140-6736(18)30953-X 29754776 PMC5984170

[38] Galon J, Mlecnik B, Bindea G, Angell HK, Berger A, Lagorce C, Lugli A, Zlobec I, Hartmann A, Bifulco C, Nagtegaal ID, Palmqvist R, Masucci GV, et al. Towards the introduction of the ‘Immunoscore’ in the classification of Malignant tumours. J Pathol. 2014; 232:199–209. 10.1002/path.4287 24122236 PMC4255306

[39] Jang JE, Hajdu CH, Liot C, Miller G, Dustin ML, Bar-Sagi D. Crosstalk between regulatory T cells and tumor-associated dendritic cells negates anti-tumor immunity in pancreatic cancer. Cell Rep. 2017; 20:558–71. 10.1016/j.celrep.2017.06.062 28723561 PMC5649374

[40] Wang TT, Zhao YL, Peng LS, Chen N, Chen W, Lv YP, Mao FY, Zhang JY, Cheng P, Teng YS, Fu XL, Yu PW, Guo G, et al. Tumour-activated neutrophils in gastric cancer foster immune suppression and disease progression through GM-CSF-PD-L1 pathway. Gut. 2017; 66:1900–11. 10.1136/gutjnl-2016-313075 28274999 PMC5739867

[41] Sica A, Mantovani A. Macrophage plasticity and polarization: in vivo veritas. J Clin Invest. 2012; 122:787–95. 10.1172/JCI59643 22378047 PMC3287223

[42] Zhang JX, Song W, Chen ZH, Wei JH, Liao YJ, Lei J, Hu M, Chen GZ, Liao B, Lu J, Zhao HW, Chen W, He YL, et al. Prognostic and predictive value of a microRNA signature in stage II colon cancer: a microRNA expression analysis. Lancet Oncol. 2013; 14:1295–306. 10.1016/S1470-2045(13)70491-1 24239208

[43] Gui J, Li H. Penalized cox regression analysis in the high-dimensional and low-sample size settings, with applications to microarray gene expression data. Bioinformatics. 2005; 21:3001–08. 10.1093/bioinformatics/bti422 15814556

[44] Tibshirani R. The lasso method for variable selection in the cox model. Stat Med. 1997; 16:385–95. 10.1002/(sici)1097-0258(19970228)16:4<385::aid-sim380>3.0.co;2-3 9044528

[45] Gordon S, Martinez FO. Alternative activation of macrophages: mechanism and functions. Immunity. 2010; 32:593–604. 10.1016/j.immuni.2010.05.007 20510870

[46] Mosser DM, Edwards JP. Exploring the full spectrum of macrophage activation. Nat Rev Immunol. 2008; 8:958–69. 10.1038/nri2448 19029990 PMC2724991

[47] Galdiero MR, Garlanda C, Jaillon S, Marone G, Mantovani A. Tumor associated macrophages and neutrophils in tumor progression. J Cell Physiol. 2013; 228:1404–12. 10.1002/jcp.24260 23065796

[48] Martínez VG, Rubio C, Martínez-Fernández M, Segovia C, López-Calderón F, Garín MI, Teijeira A, Munera-Maravilla E, Varas A, Sacedón R, Guerrero F, Villacampa F, de la Rosa F, et al. BMP4 induces M2 macrophage polarization and favors tumor progression in bladder cancer. Clin Cancer Res. 2017; 23:7388–99. 10.1158/1078-0432.CCR-17-1004 28928159

[49] Chen Y, Zhang S, Wang Q, Zhang X. Tumor-recruited M2 macrophages promote gastric and breast cancer metastasis via M2 macrophage-secreted CHI3L1 protein. J Hematol Oncol. 2017; 10:36. 10.1186/s13045-017-0408-0 28143526 PMC5286803

[50] Marsoni S and International Multicenter Pooled Analysis of Colon Cancer Trials Investigators. Efficacy of adjuvant fluorouracil and leucovorin in stage B2 and C colon cancer. International Multicenter Pooled Analysis of Colon Cancer Trials Investigators. Semin Oncol. 2001; 28:14–9. 10.1053/sonc.2001.19723 11273584

[51] Mamounas E, Wieand S, Wolmark N, Bear HD, Atkins JN, Song K, Jones J, Rockette H. Comparative efficacy of adjuvant chemotherapy in patients with dukes’ B versus dukes’ C colon cancer: results from four national surgical adjuvant breast and bowel project adjuvant studies (C-01, C-02, C-03, and C-04). J Clin Oncol. 1999; 17:1349–55. 10.1200/JCO.1999.17.5.1349 10334518

[52] Veselka J, Faber L, Liebregts M, Cooper R, Januska J, Kashtanov M, Dabrowski M, Hansen PR, Seggewiss H, Hansvenclova E, Bundgaard H, Ten Berg J, Stables RH, Jensen MK. Short- and long-term outcomes of alcohol septal ablation for hypertrophic obstructive cardiomyopathy in patients with mild left ventricular hypertrophy: a propensity score matching analysis. Eur Heart J. 2019; 40:1681–87. 10.1093/eurheartj/ehz110 31152553

[53] Hara K, Takeda A, Tsurugai Y, Saigusa Y, Sanuki N, Eriguchi T, Maeda S, Tanaka K, Numata K. Radiotherapy for Hepatocellular Carcinoma Results in Comparable Survival to Radiofrequency Ablation: A Propensity Score Analysis. Hepatology. 2019; 69:2533–2545. 10.1002/hep.30591 30805950

[54] Irizarry RA, Hobbs B, Collin F, Beazer-Barclay YD, Antonellis KJ, Scherf U, Speed TP. Exploration, normalization, and summaries of high density oligonucleotide array probe level data. Biostatistics. 2003; 4:249–64. 10.1093/biostatistics/4.2.249 12925520

[55] Ali HR, Chlon L, Pharoah PD, Markowetz F, Caldas C. Patterns of immune infiltration in breast cancer and their clinical implications: a gene-expression-based retrospective study. PLoS Med. 2016; 13:e1002194. 10.1371/journal.pmed.1002194 27959923 PMC5154505

[56] Goeman JJ. L1 penalized estimation in the cox proportional hazards model. Biom J. 2010; 52:70–84. 10.1002/bimj.200900028 19937997

[57] Deb S, Austin PC, Tu JV, Ko DT, Mazer CD, Kiss A, Fremes SE. A review of propensity-score methods and their use in cardiovascular research. Can J Cardiol. 2016; 32:259–65. 10.1016/j.cjca.2015.05.015 26315351

[58] Bünger R, Mallet RT. Metabolomics and receiver operating characteristic analysis: a promising approach for sepsis diagnosis. Crit Care Med. 2016; 44:1784–85. 10.1097/CCM.0000000000001795 27525998 PMC4988321

[59] Kang J, D’Andrea AD, Kozono D. A DNA repair pathway-focused score for prediction of outcomes in ovarian cancer treated with platinum-based chemotherapy. J Natl Cancer Inst. 2012; 104:670–81. 10.1093/jnci/djs177 22505474 PMC3341307

[60] Vickers AJ, Elkin EB. Decision curve analysis: a novel method for evaluating prediction models. Med Decis Making. 2006; 26:565–74. 10.1177/0272989X06295361 17099194 PMC2577036

[61] Vickers AJ, Cronin AM, Elkin EB, Gonen M. Extensions to decision curve analysis, a novel method for evaluating diagnostic tests, prediction models and molecular markers. BMC Med Inform Decis Mak. 2008; 8:53. 10.1186/1472-6947-8-53 19036144 PMC2611975

