# Supplementary material for: A 12-immune cell signature to predict relapse and guide chemotherapy for stage II colorectal cancer
Source: Aging (Albany NY). 2020 Aug 27;12(18):18363–83. doi: 10.18632/aging.103707 (PMC7585080; doi:10.18632/aging.103707)
Supplement: Supplementary Figures [file aging-12-103707-s002..pdf]

SUPPLEMENTARY FIGURES

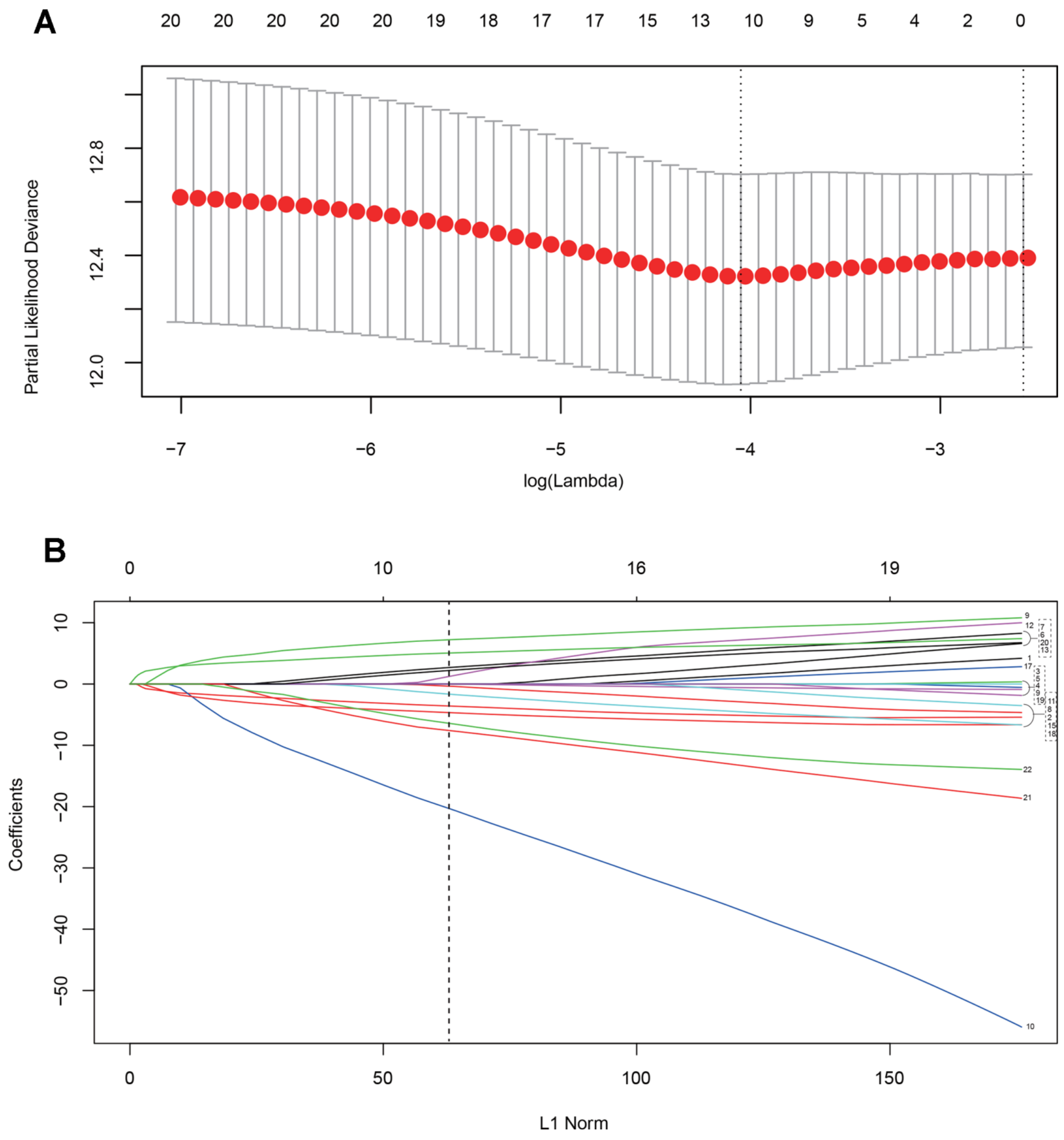

Supplementary Figure 1. Cross-validation for tuning parameter selection in the LASSO model. The point plot of partial likelihood deviance versus log (lambda). The red dotted line represents ten-fold cross-validation curve. The solid vertical lines are partial likelihood deviance standard error (SE). The dotted vertical lines are drawn at the optimal values by minimum criteria and 1-SE criteria (**A**) The trajectory of each candidate immune cell's coefficient in CRC was observed in the LASSO coefficient profiles with the changing of the lambda (**B**).

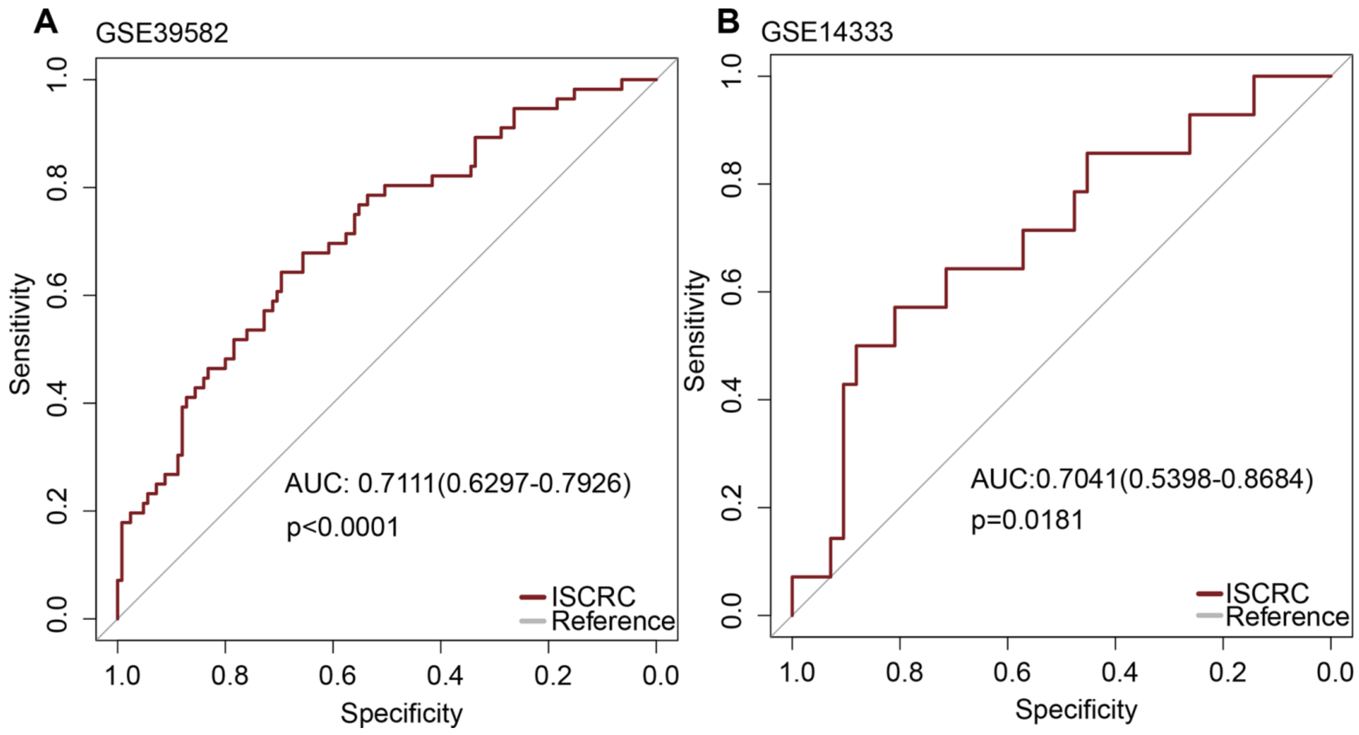

Supplementary Figure 2. Receiver operating characteristic (ROC) analysis of the ISCRC model. The sensitivity and specificity of the recurrence prediction in GSE39582 (N=253) (**A**) and GSE14333 (N=86) (**B**).

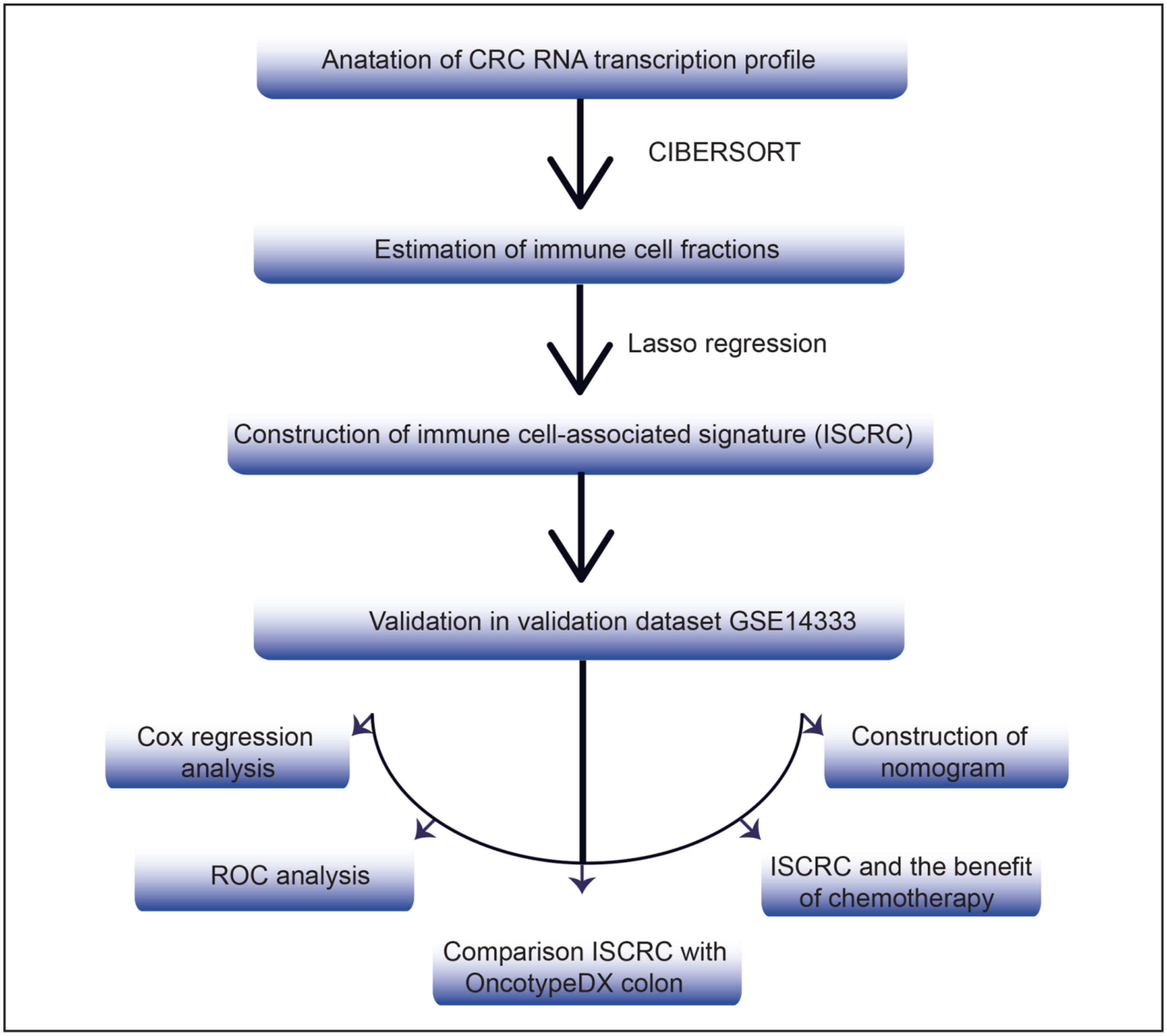

Supplementary Figure 3.The workflow of this study. The orders of analyses to generate the ISCRC model and assess its prognostic value in stage II CRC, etc.
