# Supplementary material for: A 12-immune cell signature to predict relapse and guide chemotherapy for stage II colorectal cancer
Source: Aging (Albany NY). 2020 Aug 27;12(18):18363–83. doi: 10.18632/aging.103707 (PMC7585080; doi:10.18632/aging.103707)
Supplement: Supplementary Tables [file aging-12-103707-s001..pdf]

SUPPLEMENTARY TABLES

Supplementary Table 1. Immune cells significantly associated with the disease-free survival in the training cohort (N=253).

Immune cells	Coefficent
B cells momory	-3.39085
T cells CD4 memory activated	2.333988
T cells follicular helper	-0.10681
T cells regulatory Tregs	6.994373
T cells gamma delta	-18.5763
NK cells actibated	0.249715
Macrophages M1	-4.44003
Macrophages M2	4.89173
Dendritic cells activated	-1.24627
Mast cells activated	1.840128
Eosinophils	-7.01601
Neutrophils	-5.69189

Supplementary Table 2. Calculation of prognostic indexes.

CALCULATION OF PROGNOSTIC INDEX FOR 12-IMMUNE CELL SIGNATURE (ISCRC)

The calculation of the immune risk score for individual patients was based on the multivariate model including all twelve immune cells as shown in table S1. Firstly, through LASSO Cox regression analysis, we have identified 12 prognostic immune cells and their associated coefficients. Then, multiply the fraction of each immune cell by its associated coefficient to generate the value for each immune cell. At last, sum all the values of 12 immune cells to get the immune risk score for each patient. The formula was as follows:

Immune risk score = (-3.39085 × fraction of B cells momory)+(2.333988 × fraction of T cells CD4 memory activated)+(-0.10681 × fraction of T cells follicular helper)+(6.994373 × fraction of T cells regulatory Tregs)+(-18.5763 × fraction of T cells gamma delta)+(0.249715 × fraction of NK cells actibated) +(-4.44003 × fraction of Macrophages M1) +(4.89173 × fraction of Macrophages M2) +(-1.24627 × fraction of Dendritic cells activated) +(1.840128 × fraction of Mast cells activated)+ (-7.01601 × fraction of Eosinophils) + (-5.69189 × fraction of Neutrophils)

ALGORITHMS TO CALCULATE RISK SCORES FOR ONCOTYEDX

Recurrence Risk score is calculated using the prespecified genes and algorithm [1, 2]:

The O'Connell Recurrence Risk (RS) score is composed of 12 genes among which 5 reference genes and 7 genes associated to recurrence.

For the reference genes, when several probe set were possible, the less variant one was selected.

For the other genes, data were median gene centered and aggregated by mean if several probe sets were available.

Then the recurrence genes intensities for each sample were subtracted by the mean of the reference gene per sample and the formula was applied for each sample. RSu = 0·15* mean(BGN,FAP,INHBA)-0·3*mean(MKI67,MYC,MYBL2)+ 0·15* GADD45B)

This score was then rescaled RS=44*(RSu+0.82).

REFERENCES

1. Yothers G, O'Connell MJ, Lee M, Lopatin M, Clark-Langone KM, Millward C, Paik S, Sharif S, Shak S, Wolmark N. Validation of the 12-gene colon cancer recurrence score in NSABP C-07 as a predictor of recurrence in patients with stage II and III colon cancer treated with fluorouracil and leucovorin (FU/LV) and FU/LV plus oxaliplatin. J Clin Oncol. 2013; 31:4512–9. https://doi.org/10.1200/JCO.2012.47.3116 PMID:24220557

2. Gray RG, Quirke P, Handley K, Lopatin M, Magill L, Baehner FL, Beaumont C, Clark-Langone KM, Yoshizawa CN, Lee M, Watson D, Shak S, Kerr DJ. Validation study of a quantitative multigene reverse transcriptase-polymerase chain reaction assay for assessment of recurrence risk in patients with stage II colon cancer. J Clin Oncol. 2011; 29:4611–9. https://doi.org/10.1200/JCO.2010.32.8732 PMID:22067390

Supplementary Table 3. propensity score matching analysis.

GSE39852 dataset			
Before Matching	Stratified by chemotherapy		SMD*
	0	1	
N	198	54	
Sex (mean (SD))	1.40 (0.49)	1.41 (0.50)	0.007
Age at diagnosis (mean (SD))	69.78 (12.62)	59.91 (10.47)	0.851
Tumor location (mean (SD))	1.43 (0.50)	1.22 (0.42)	0.451

After Matching	Stratified by chemotherapy		SMD*
	0	1	
N	46	27	
Sex (mean (SD))	1.22 (0.42)	1.19 (0.40)	0.079
Age at diagnosis (mean (SD))	66.39 (7.30)	65.07 (8.28)	0.169
Tumor location (mean (SD))	1.11 (0.31)	1.11 (0.32)	0.008

GSE14333 dataset			
Before Matching	Stratified by chemotherapy		SMD*
	0	1	
N	64	21	
Sex (mean (SD))	1.42 (0.50)	1.62 (0.50)	0.396
Age at diagnosis (mean (SD))	69.06 (13.20)	62.86 (11.28)	0.505
Tumor location (mean (SD))	1.53 (0.50)	1.52 (0.51)	0.015

After Matching	Stratified by chemotherapy		SMD*
	0	1	
N	8	8	
Sex (mean (SD))	1.62 (0.52)	1.62 (0.52)	<0.001
Age at diagnosis (mean (SD))	71.75 (8.26)	70.62 (8.52)	0.134
Tumor location (mean (SD))	1.75 (0.46)	1.75 (0.46)	<0.001

*SMD is short for Standardized Mean Differences.
